# Paediatric fibrosarcoma treatment

**DOI:** 10.3332/ecancer.2023.1608

**Published:** 2023-10-02

**Authors:** Adrián Cano-Padilla, Augusto Ramírez, Paola Cervantes-Rivera, Rosalba Bellido-Magaña, Gilberto Flores-Vargas, Nicolás Padilla-Raygoza

**Affiliations:** 1 General Surgery, León General Hospital, Guanajuato State Public Health Institute, Leon, CP 37672, México; 2 Department of Thoracic and Vascular Surgery, León General Hospital, Guanajuato State Public Health Institute, Leon, CP 37672, México; 3 Department of Plastic and Reconstructive Surgery, León General Hospital, León Institute of Public Health, Guanajuato State Public Health Institute, Leon, CP 37672, México; 4 Department of Paediatric Oncology, León General Hospital, Guanajuato State Public Health Institute, Leon, CP 37672, México; 5 Department of Research and Technological Development, Director of Teaching and Research, Guanajuato State Public Health Institute, Guanajuato, CP 36000, México

**Keywords:** children, fibrosarcoma, soft tissue, surgery

## Abstract

**Introduction:**

Soft tissue sarcomas make up 7%–15% of childhood solid tumours. The aetiology of this disease is unknown. It is a fast-growing, painless tumour; histologically similar to adult fibrosarcoma, but having a lesser risk of metastasis and a better prognosis. The treatment is aimed towards localised intervention; complete surgical resection is the appropriate treatment as long as it can be performed.

**Case report:**

An 11 years old female was referred for resection of a soft tissue tumour on the right elbow with significant peripheral vascularisation. Tumour resection was scheduled, with the placement of a partial thickness skin graft, and a piece was sent to pathology; a histological type consistent with paediatric fibrosarcoma was obtained with margins less than 1 mm from the lesion. Therefore, the patient was referred to the paediatric oncology unit. Further studies with positron emission tomography were requested, in which no evidence of macroscopic anatomy-metabolic tumour activity was found. Subsequently, treatment was started by paediatric oncology with 2 sessions of chemotherapy and 20 sessions of radiotherapy with sufficient progress; finally, assessment by plastic and reconstructive surgery was performed and an adequate quality of graft was observed, without the need for any other intervention by their service.

**Conclusion:**

The involvement of the vascular surgeon in performing the tumour resection permitted the preservation of the best circulation to the extremity, thereby, avoiding amputation. The difficult decision made by the reconstructive surgeon to place a partial thickness graft over the surgical site, and to start radiotherapy/chemotherapy by paediatric oncology, were key to the success in achieving the patient’s satisfactory progress.

## Introduction

Malignant neoplasias in children and adolescents are uncommon but represent an important impact on health in these age groups [1]. Despite the gradual reduction in mortality due to neoplasia, cancer still represents the primary cause of death by disease among children and adolescents [2]. Soft tissue sarcomas make up 7%–15% of paediatric solid tumours. Fibrosarcoma is the second most common tumour, after rhabdomyosarcoma, representing 11% [3].

The aetiology of this disease is unknown [7]. Clinically, it is a fast-growing, painless tumour, and other symptoms are localised spreading or compression of adjacent structures [4].

Paediatric fibrosarcoma is related to cytogenetic translocation t(12;15)(p13;q25), which leads to the fusion of genes ETV6-NTRK3 [5]. Furthermore, other forms of translocation have also been observed, including EML4-NEML4-NTRK3, TPM3-NTRK1, TRK3, TPM3-NTRK1, LMNA-NTRK1 y LMNA-NTRK1 and BRAF intragenic deletions [6].

Regarding paraclinical studies: computerised axial tomography and/or magnetic resonance assist in determining the size of the tumour, the spread and invasion into adjacent organs [7].

Histologically it is similar to the fibrosarcomas that appear in adults but differs in that. It is associated with a lower risk of metastasis, and a better prognosis in general [8]. Lymphatic involvement is infrequent at less than 10%, similar to the presence of distant metastasis [9].

The treatment is aimed at local control; complete surgical resection is the indicated treatment as long as it can be performed. In case of incomplete resection or inadequate margins, consolidation radiotherapy is carried out [10]. Adjuvant chemotherapy is uncertain and is not regularly utilised due to the low incidence of distant metastasis [11].

## Case report

An 11-year-old female was admitted to the vascular surgery service of León General Hospital, referred by Irapuato General Hospital for a tumour on the right elbow observed by her mother 6 months before her assessment. Nuclear magnetic resonance (NMR) of the right thoracic extremity was requested and a soft tissue tumour with significant peripheral vascularisation was found.

**Figure 1.**
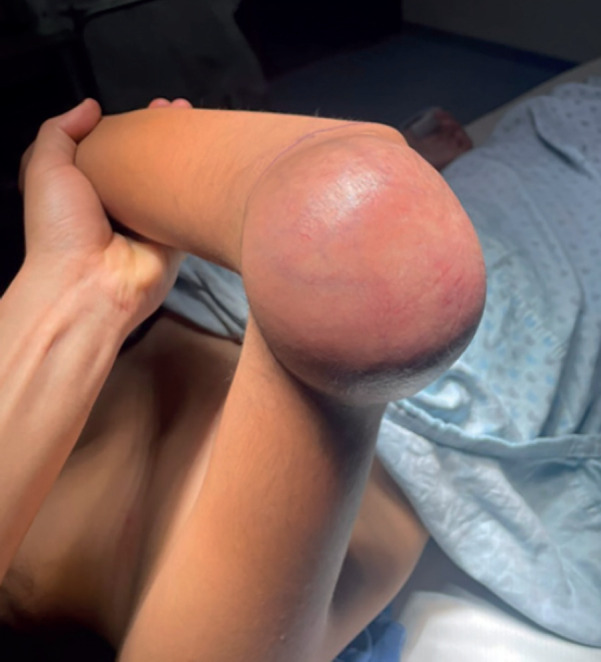
Tumefaction on the right elbow. 10 × 15 cm right elbow tumour before surgical intervention. Source: Clinical record.

Figure 1 shows a photo of the tumour on the right elbow before surgical intervention.

**Figure 2.**
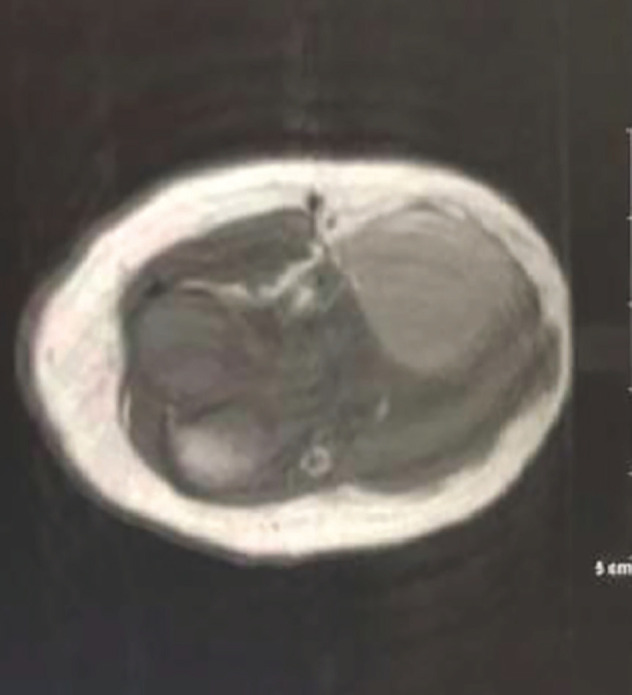
NMR of the right elbow. Source: Clinical record.

Figure 2 shows the NMR image of the tumour, showing vascularisation and non-involvement of the bone tissue.

NMR with vascularised tumour growth in the right elbow without any apparent bone involvement.

**Figure 3.**
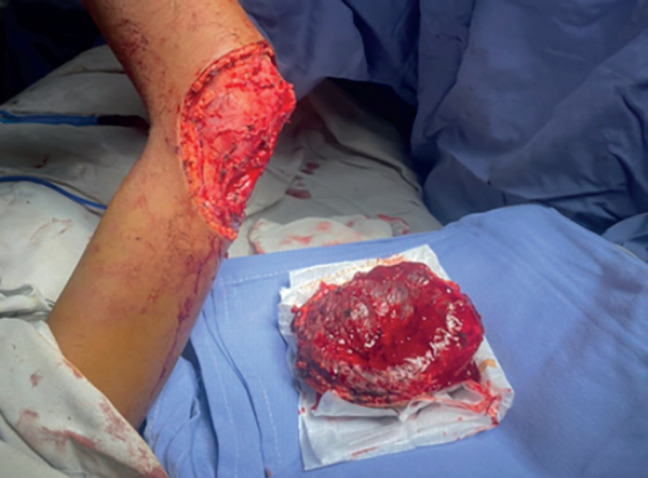
Excision of tumour on right elbow. Source: Clinical record.

**Figure 4.**
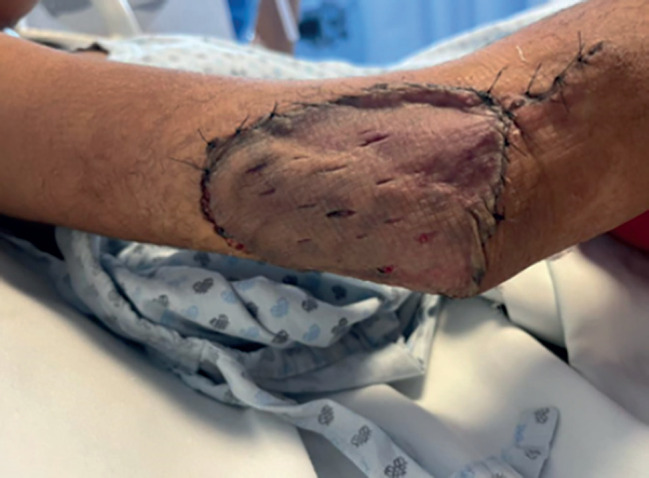
Partial thickness graft on the right elbow. Source: Clinical record.

Excision of the tumour on the right elbow was scheduled, and a vascular tumour of approximately 10 × 15 cm was found; soft consistency, not adhered to deeper levels nor bone structures, with several alimentary vessels; the placing of a partial thickness skin graft of approximately 15 × 15 cm taken from the right thigh was performed (Figures 3 and 4).

In Figure 3, the excised tumour is shown, showing significant vascularisation and no bone involvement.

Resected tumour, showing significant vascularisation with no bone involvement, as well as the 15 × 10 cm surgical site.

In Figure 4, the skin graft placed on the right elbow is shown. This graft was obtained from the thigh on the same side of the body.

A partial thickness graft was placed on the right elbow and obtained from the ipsilateral thigh.

**Figure 5.**
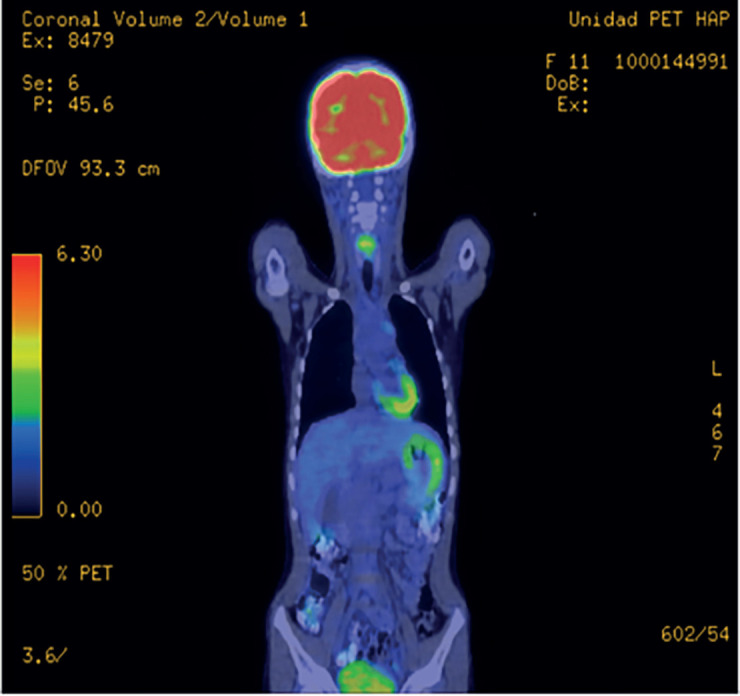
Coronal PET CT. Source: Clinical record.

**Figure 6.**
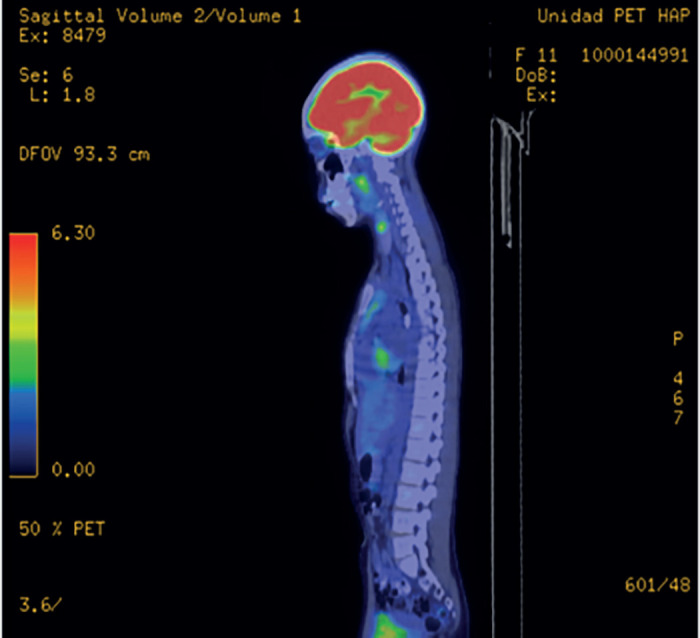
Sagittal PET CT. Source: Clinical record.

A piece was sent for pathology which showed a histological type consistent with paediatric fibrosarcoma with margins of less than 1 mm from the lesion. The patient was sent to the paediatric oncology unit, where further studies via positron emission tomography (PET CT) were requested, in which no evidence of macroscopic anatomo-metabolic tumour activity were found, with hypermetabolic changes of physiological origin in the mediastinum and post-surgical inflammation in the right elbow (Figures 5 and 6).

In Figure 5, the PET CT does not show evidence of tumour activity.

Coronal PET CT without evidence of macroscopic anatomo-metabolic tumour activity, with hypermetabolic changes of physiological origin in the mediastinum.

The PET CT sagittal cross-section in Figure 6 does not show tumour activity either.

Sagittal PET CT without evidence of macroscopic anatomo-metabolic tumour activity, with hypermetabolic changes of physiological origin in the mediastinum.

**Figure 7.**
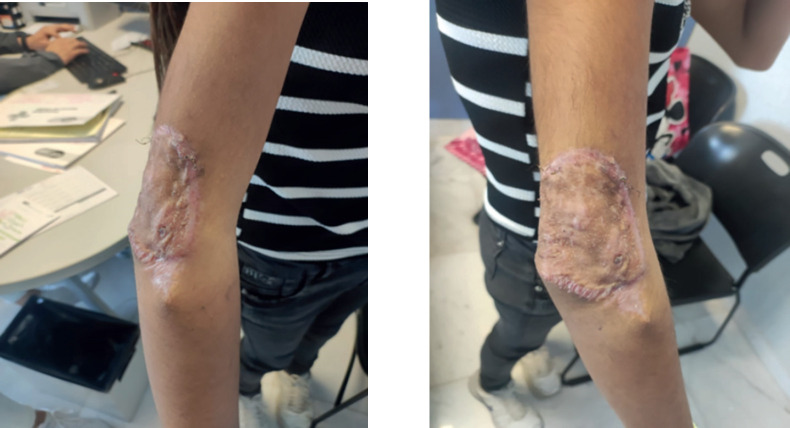
Skin graft examination. Source: Clinical record.

Subsequently, treatment was started by paediatric oncology with two sessions of chemotherapy with a weekly 1.5 mg/m^2^/dose of vincristine, as well as 20 sessions of post-operative radiotherapy of 50 Gy in 20 fractions to the surgical site in conformal modality with adequate progress. Finally, assessment by plastic and reconstructive surgery was performed and an adequate quality of graft was observed, without the need for any other intervention by that service (Figure 7).

In Figure 7, the state of the skin graft after the chemotherapy and radiotherapy sessions is shown.

The patient was examined at an external reconstructive surgery consultation to assess the skin graft after the chemotherapy and radiotherapy sessions.

The patient was examined at an external reconstructive surgery consultation to assess skin graft after the chemotherapy and radiotherapy sessions.

## Discussion

The involvement of the vascular surgeon is of great importance in carrying out the tumour excision to maintain the best circulation possible to the extremity and, in turn, to avoid its amputation. In this case, a complete removal of the fibrosarcoma, which was found not to be adhered to deep levels, was performed.

After the resection of the tumour, in the same surgical session, an autologous partial-thickness skin graft obtained from the patient’s ipsilateral thigh was placed. Initially, placement of an autologous full-thickness graft was planned, considering that it is the most common type of graft used in these cases and to achieve a greater rate of acceptance after radiotherapy sessions, but the patient’s slenderness made it impossible to obtain said graft; therefore, a partial thickness grated was selected. After the chemotherapy and radiotherapy sessions, the condition of the graft was assessed and adequate quality was observed; therefore, it was not necessary to intervene again [12].

Patients with residual disease following resection who receive adjuvant therapy progress well (5 years survival, 76%). A positive post-surgery margin does not always indicate a poor outcome [13].

Neoadjuvant chemotherapy should be used for extensive lesions when a complete surgical removal during an initial procedure is not possible or would result in amputation. Neoadjuvant chemotherapy has been recommended to reduce the risk of both local recurrence and metastasis. The role of adjuvant chemotherapy after initial surgery is less clearly defined. The most commonly used drugs are vincristine, cyclophosphamide, actinomycin-D and doxorubicin [13].

It has been reported that radiotherapy had significantly better local control in patients with microscopic residual disease who received post-operative radiotherapy [14].

Factors associated with local recurrence include post-operative microscopic residual disease, primary intra-abdominal tumour and the omission of adjuvant radiotherapy. Paediatric fibrosarcoma, despite its similarity with the adult variant, tends to be much less aggressive with its limited metastasis potential [14].

It was a difficult decision taken by plastic and reconstructive surgery to place a partial thickness graft, given that in the majority of cases, the use of chemotherapy and radiotherapy favours its rejection, but it was decided to take the risk to place it due to the patient’s general adequate condition, and satisfactory results were obtained. Finally, the patient will be routinely monitored by paediatric oncology to confirm the absence of disease.

## Conclusion

The timely recruitment of patients with suspicion of any type of neoplasia is vital for their prognosis. In this case, the paediatric fibrosarcoma progressed quickly, which resulted in a referral to a suitable secondary care centre, where timely procedures were carried out for the well-being of the patient.

The teamwork by vascular surgery, reconstructive surgery, and paediatric pathology and oncology resulted in the patient’s adequate progress as a result of being cared for at a secondary-level institution, for which we take into account that they have the necessary tools to care for this patient and others.

The physical and emotional impact of the diagnosis, for the patient as well as their family, was devastating, but the timely attention and well-established treatment protocols led to a good result. Therefore, all health personnel is encouraged to emphasise the importance of quick attention to neoplastic processes, and finding a corresponding specialist for each case.

## Conflicts of interest

The authors declare that they have no conflicts of interest.

## Funding

The Guanajuato State Institute of Public Health has financed the Publication’s Open Access costs. In Guanajuato State, the healthcare for the state’s population without Social Security (entitlement) is free.

## Informed consent

The minor’s parents signed informed consent for their medical care at León General Hospital.

Afterward, informed consent was requested from the minor, to publish their surgical data and images, highlighting that their personal data would not be published.

## Author contributions

Adrián Cano-Padilla, Augusto Ramírez-Solís, Paola Cervantes-Rivera, Rosalba Bellido-Magaña, collaborated in collecting data and patient treatment. Gilberto Flores-Vargas y Nicolás Padilla-Raygoza edited the final version of the case reports.
